# Roadmap to Indian Psychiatry

**DOI:** 10.4103/0019-5545.55083

**Published:** 2009

**Authors:** E. Mohandas

**Affiliations:** Chief consultant, Elite Mission Hospital, Koorkkenchery, Trissur, Kerala

## INTRODUCTION

“*We are responsible for what we are, and whatever we wish ourselves to be, we have the power to make ourselves. If what we are now has been the result of our own past actions, it certainly follows that whatever we wish to be in future can be produced by our present actions; so we have to know how to act*.”

- Swami Vivekananda

With all its alluring contrasts and remarkable features, India has a grand heritage of 34,000 years. Down the traditional lane, it has evolved into a pluralistic, multilingual, and multiethnic society. It is quite striking that while India is the second-most populous country, it has the largest democracy in the world. The second fastest growing economy and the third largest military force are also her golden quills. Seventy-four per cent of the 1.14 billion population of India live in rural areas. India has 28 states, seven union territories, 612 districts, and 6, 38,365 villages. The fact that there are 22 official languages in a single country is ample evidence for the heterogeneous nature of the society it represents. Having a literacy rate of about 64.8%, India has 80 million internet users. With all this, I do not mean to say that India is not without its lacunae. Even while India is the world's twelfth largest economy, we cannot close our eyes to the grim truth that 22% of the population exists below the poverty line.[1–3]

With regard to the academic scene, India is proud to have 289 institutions providing undergraduate medical training (196 MCI recognized, 77 MCI permitted, and 16 in the “danger zone”). The postgraduate training in psychiatry includes Doctor of Medicine (MD) (83 centers, 159 seats), Diploma in Psychological Medicine (DPM) (46 centers, 107 seats), and Diplomate of National Board (22 centers, 36 seats). Strikingly, 25% of the medical colleges in India do not have a Psychiatry Department.[4–6] Apparently, there are only around 4000 psychiatrists in India to serve the five crore mentally ill population currently.

## MENTAL HEALTH SCENARIO IN INDIA

Obviously, in a vast country like India, the threat posed by the psychiatric and behavioral disorders is just inexplicable. A meta-analysis of 13 epidemiological studies consisting of 33,572 persons reported a total morbidity of 58.2 per 1000. Another meta-analysis of 15 epidemiological studies reported a total morbidity of 73 per 1000.The saddest aspect is that the bulk of the affected falls in the 15 to 45 year age group. The existing facilities in the country fall short of the required norms, which makes the situation still worse. The number of psychiatric beds in the country is only about 0.2 per 1, 00,000 population and there are only two psychiatrists per 10 lakh population. The major share of psychiatric facilities lies with the government sector (especially mental hospitals), which is centered on certain areas of particular states. The psychiatric services have not yet been integrated into the primary health care system and this leaves large populations in dire need of such facilities, with no hope of effective treatment. Therefore, they seek help from the private sector and there are no clear policies regarding treatment of the mentally ill in the private sector. A significant population in India cannot afford private hospital care and the insurance system in the country is in its infancy. The rehabilitation of psychiatric patients is also given little importance in the existing mental health framework. The integration of psychiatric services to primary care needs a public–private partnership to enable comprehensive mental health care.[7–10]

## INDIAN PSYCHIATRIC SOCIETY EVOLUTION

Our society sprouted from the Indian Association for mental hygiene founded in 1929 by Berkeley Hill. In 1935, the Indian division of the Royal Medico-Psychological Association (RM-PA) was formed, due to the efforts of Dr. Banarasi Das. Thanks to the efforts of Dr. Nagendra Nath De, Major R. B. Davis, and Brigadier T. A. Munro, the association gained its new name, the Indian Psychiatric Society (IPS) on 7 January, 1947. The rules and regulations were framed by the eminent Psychiatrists of that period (Dhunjibhoy, Rosie, Kenton, Llyodo, Masani, Shah, Johnson, Govindaswamy, and Kak). The first annual meeting held on 2 January, 1948, at Patna, was presided by N.N. De.[11,12] The society has grown into a group of 2000 Fellows (right of franchise) and many ordinary members. The activities of IPS are also delegated to state branches coming under five zones. The objectives of the society are very comprehensive.[13]

- Promote and advance the subject of Psychiatry and allied sciences in all their different branches.
- Promote the improvement of the mental health of people and mental health education.
- Promote prevention, control, treatment, and relief of all psychiatric disabilities.
- Formulate and advice on the standards of education and training for medical and auxiliary personnel in psychiatry, and recommend adequate teaching facilities for the purpose.
- Promote research in the field of psychiatry and mental health. Propagate the principles of psychiatry and current development in psychiatric thought.
- Deal with any matters relating to mental health concerning the country and conduct all other things as are cognate to the subjects of the Indian Psychiatric Society.
- Safeguard the interest of Psychiatrists and fellow professionals in India.
- Promote the ethical standards in the practice of psychiatry in India.

It is worthwhile to introspect on our achievements and deficits.

The achievements include academic updates as part of professional development, in the form of publications like Indian Disability Evaluation and Assessment scale (IDEAS) and Clinical Practice Guidelines, and the Indian Journal of Psychiatry. Some efforts have been carried out in mental health literacy and community service strategies. Although psychiatrists are involved in NMHP (National Mental Health Program) and DMHP (District Mental Health Program), the society's involvement as a stakeholder is still not appreciated. The same is true for other mental health policies and programs.

IPS should

1. Be a stakeholder in mental health policy matters.
2. Have its publications on mental health issues.
3. Try to get its journal indexed in international databases.
4. Actively involve in the initiative to have a Mental Health Website by the Health Ministry.
5. Address social issues, conduct mental health literacy programs, and open free mental health service outlets in an organized and planned manner, if possible.
6. Have an advocacy team to facilitate the link between the society and officialdom, in matters such as undergraduate psychiatric training, decriminalization of ‘attempted suicide,’ and ‘homosexuality’, better functioning of DMHP, and refining the Mental Health Act.

## THE VISION

### 1. Reaching the unreached

The rural population, comprising of about 74%, is beset with multiple disadvantages. High population growth rate, agrarian form of economy, primitive agricultural practices, illiteracy, ignorance, unemployment, underemployment, caste-based politics, urban rural divide, social iniquity, and discrimination, account for 22.15% of the population that remain below the poverty line. “State of the World Population 2007” report comments on the rapid shift of rural population to the cities by 2008. This ‘pseudo-urbanization’ may lead to shortage of resources in cities leading to ‘urban poverty’. Relationship between poverty and poor mental health has been well studied and stated. The World Health Organization report on mental health states ‘Mental disorders occur in persons of all genders, ages, and backgrounds. No group is immune to mental disorders, but the risk is higher among the poor, homeless, unemployed, and persons with low education’. Poverty, unemployment, poor education, and poor nutrition may pave the way for maladaptive behavior, depressive illness, and broken families. The vicious cycle of poverty breeding mental dysfunction may culminate in substance use, domestic violence, and antisocial behavior. The pathological family atmosphere may cast a negative impact on a child's mental health.[14–16]

Ignorance, illiteracy, ‘myth understanding’, poor access to psychiatric services, and fractured community care / support necessitate the need for mental health literacy, Psychoeducation, proper immunization, improved nutritional care, and better mental health service delivery in the rural population. This may be facilitated by IPS in collaboration with NRHM (National Rural Health Mission) and DMHP.

### 2. Rehabilitation

Psychiatric rehabilitation facilities do not satisfy even the adequate requirements in many states across India. There is a polarization toward South India, especially Kerala, Karnataka, and Tamil Nadu, in the psychosocial rehabilitation map. Training facilities exist at NIMHANS, Richmond Fellowship Society of India at Bangalore, and SCARF at Chennai. Many NGOs are involved in rehabilitation practices, especially in substance use disorders, human immunodeficiency virus (HIV), dementia, and schizophrenia. However, IPS has not done enough in this area except for formulation of IDEAS.

It is quite appropriate and appreciable to have a status report on the psychiatric rehabilitation facilities, and initiate skill development in psychiatric rehabilitation under the umbrella of IPS.

### 3. Research

The research output from India concentrates mainly on epidemiology and service delivery. The database on drug abuse research has been mainly from AIIMS (New Delhi) and NIMHANS (Bangalore). Psychiatric genetic research is another domain, again from New Delhi and Bangalore. Biological psychiatry research initiated from Lucknow has now shifted, mainly to Bangalore. The priorities in research on mental health have been outlined elsewhere.[17–18] Research publications in the international database during the last decade mainly reflect on the studies generated from selected teaching institutions in India — NIMHANS (Bangalore), PGI (Chandigarh), AIIMS (New Delhi), RML hospital (New Delhi), KGMC (Lucknow), IHBAS (New Delhi), and CIP (Ranchi). CMC (Vellore) deserves a special mention for its collaboration with the Cochrane Database.

There are efforts being made to conduct and pursue research in other teaching institutions and private psychiatric centers. However, the limiting factors include,

1. Lack of proper training in research methodology.
2. Lack of motivation.
3. Lack of infrastructure.
4. Lack of skill in writing a research article.
5. Lack of funding.

**Table:** Central budget allocation for training and research — 2008-2009

Center	Budget Allocation (in crores)
AIIMS	452
PGI	183
JIPMER	128
Lady Hardinge Medical College and Smt. Sucheta Kripalani Hospital, New Delhi	99
NIMHANS	68
RML	10.8
Others	10.9

**Table:** Central budget allocation for research — 2008-2009

Center/Activity	Budget allocation (in crores)
ICMR	356 (How much for mental health research?)
Promotion, coordination and development of basic, applied, and clinical research	50
Inter-sectorial coordination in medical, biomedical, and health research	19
Advanced training in research in medicine and health	5.5
International cooperation in medical and health research	10
Matters relating to epidemics, natural calamities, and development of tools to prevent outbreaks	5
Matters relating to scientific societies and associations, charitable and religious endowments in medicine, and health research areas	2
Other health research schemes	1
Provision for projects/schemes of North, Eastern Areas, and Sikkim	40

Hospitals and dispensaries have separate allocation for development plans

A bird's eye view on the Central Budget allocation under the Health Ministry[19,20] can reveal why major teaching institutions have been able to churn out research data.

It would be worthwhile to look at the budget allocation for research, and propose a research agenda for mental health.

However, the state-run institutions depend upon research grants from their respective state governments. The allocation for mental health research might be negligible or almost nil, partly due to the myopic vision of the mental health planners in the ministry or due to “masterly inactivity” of the state psychiatric associations. There is a provision from the Central Ministry for upgrading selected institutions to the ‘AIIMS Model’ with an allocation of 490 crore rupees. If properly planned and executed, the barren land for research in mental health may hopefully become fertile in the near future.[19]

IPS should focus on this issue and try to facilitate research in psychiatry. The possible methods include,

1. Research methodology training to selected psychiatrists.
2. Advocacy to reallocate and channelize research grants. A core group should screen the proposals and the advocacy committee of IPS should try to impress the ministry and ICMR (Indian Council of Medical Research) to take appropriate steps. NMHP has 58 crores as its share, whereas, NRHM having 10786.25 crores as budget allocation, encompasses the National Drug De-addiction Control Program (11 crores) and Information Education and Communication (171.7 crores). The core group may try to include mental health related programs under NRHM, such as,
3. Orientation in skills in drafting a research article.
4. Young researchers group under IPS may have a positive impact.
5. The networking of the international association may render assistance to mental health research in India.

### 4. Women' issues

Forty-eight per cent of the Indian population is constituted by women. Although Indian history depicts equal rights for women in ancient India, the medieval period denied equal rights. The Indian constitution has many provisions for the welfare of women as can be learnt from article 14 (equality), article 15 (1) (no discrimination by the State), article 16 (equality of opportunity), article 39 (equal pay), article 51 (A) (e) (dignity of women), and article 42 (maternity relief). The Dowry Prohibition Act of 1961, National Policy for the Empowerment of Women in 2001, and The Protection of Women from Domestic Violence Act of 2005 (which came into force in October, 2006), are geared toward the welfare of women. Despite all this, discrimination and oppression are rampant, especially in rural areas. Less than 10% of the households are matriarchal. It has been predicted that the growth rate of crimes against women might surpass the population growth rate by 2010. Poverty, illiteracy, malnutrition, infections, and improper maternity care in rural areas might be the reason for the high rate of maternal mortality in India (second highest in the world).[21,22]

A meta-analysis of 13 epidemiological studies from the different regions of India revealed an overall prevalence rate of mental disorders in women of 64.8 per 1000.[8] Women are twice prone to develop depression compared to men. Poverty and malnutrition may have a direct link in causing depression during pregnancy and the post partum period. The hormonal milieu of the female gender may render vulnerability to emotional disorders and may influence the pharmacokinetics of drugs. Women's mental health issues have been discussed elsewhere.[8,21,22]

### 5. Physical illness in mentally ill

Several large studies show that psychiatric patients suffer a high rate of co-morbid medical illnesses. Co-morbid depression in the medically ill is often undiagnosed and untreated, leading to increased morbidity. Lack of proper training, defective perception of the psychological impact on physical disorders, improper rationalization about the somatic symptoms of depression, and a tunnel vision of medical pathology may culminate in the physician's non-recognition of the psychopathology. Co-morbid psychopathology may contribute to the therapeutic nonadherence to medical regimen. The drug—drug interaction may pose a therapeutic dilemma.[23]

Let me now focus on two specific issues relevant to the Indian context — Diabetes and HIV.

With a 40 million diabetic population, India has earned recognition as the diabetic capital of the world. The characteristics of higher glycemic response to all food items, secreting more insulin in response to glucose, and the Asian Indian Phenotype (increased insulin resistance, greater abdominal adiposity, lower adiponectin, and higher high-sensitivity C-reactive protein levels) demonstrate a higher vulnerability to develop type II diabetes, with a projected 70 million by 2025.[24–28]

A meta-analysis of 42 studies has shown clinically relevant depression in 31% of diabetics. Diabetics are twice as likely to have depression. Co-morbid depression may affect glycemic control and diabetes self-care behavior. Depression may also add to disability and decreased Quality of Life (QOL). Medication nonadherence can lead to increased morbidity and mortality. The relationship between depression and diabetes may be summarized as follows:[27,28]

1. Depression is a risk factor in the development of Type II diabetes mellitus (DM).
2. Depression increases the risk of coronary heart disease (CHD) in established DM.
3. Depression is associated with hyperglycemia and other metabolic abnormalities.
4. Depression is associated with other physical precipitants of heart disease.
5. Treatment of depression may reduce the risks of DM and its complications.

About half of the Indian population consists of adults in the sexually active age group. The National Family Health Survey conducted between 2005 and 2006, has measured HIV prevalence among the general adult population of India. The revised prevalence estimate in July 2007, suggests that around 2.5 million people in India are living with HIV. The HIV prevalence in female sex workers in India is around 5%, mainly accounted for in Maharashtra and Nagaland. HIV prevalence is high in intravenous drug users (IDU).The rates are as high as 64% in certain cities. Manipur ranks first, with six times the prevalence of Maharashtra and 20 times that of Tamilnadu. Concurrent hepatitis C/B, tuberculosis, anemia, and cellulitis complicate the management of HIV in IDU.[29–31]

The psychiatric disorders in HIV may range from adjustment disorders, depression, and anxiety states to acquired immune deficiency syndrome (AIDS) dementia. Rates of depression in Indian HIV cases range from 10 – 40%, higher than the rate reported elsewhere. However, the rates of AIDS dementia are pretty low (1 – 2%).[29]

Research in psychiatric disorders in HIV will be an area where young researchers in Psychiatry have to focus.

### 6. Nonpharmacological treatment

Training in the nonpharmacological management of psychiatric disorders is abysmally poor in India. Although very few teaching institutions impart the knowhow of cognitive behavior techniques and basic psychotherapeutic skills, a majority of the practitioners employ ‘common sense’ psychotherapy. The upswing of psychopharmacological research has provided a generation of ‘pill pushers,’ relegating the very essence of a good doctor – patient relationship and nonpharmacological management principles. It has to be remembered that a ‘psychological mattress’ is essential for a ‘pharmacological pillow’.

The time is ripe to initiate skill development in cognitive behavior therapy (CBT) and other nonpharmacological management strategies.

### 7. Child psychiatry

It is estimated that as many as one in five children and adolescents may have identifiable mental health disorders requiring treatment. Mental health disorders in children and adolescents are caused by biology, environment, or a combination of the two. A hostile, threatening or uncomfortable environment provides the breeding ground for many negative perceptions with resultant emotional and behavioral disturbances. Families and communities, working together, can help children and adolescents with mental disorders. A broad range of services is often required to meet the needs of these young people and their families.

Child psychiatry training in India is available only in very few centers. Most of the postgraduate training centers do not address psychological/psychiatric issues in a desired format, thanks to the lack of proper training in that specialty. The cry for a super specialty in child psychiatry did not impress the authorities. However, the NIMHANS model of child psychiatry specialization is a welcome step.

There is an urgent need to pool the available child psychiatrists of India and to initiate time-bound, focused, training workshops.

### 8. Geriatric psychiatry

The elderly population in our country will increase from 7.6 million in 2001 to 137 million by 2021. The feelings of loneliness along with the natural age-related decline in physical and physiological functioning are catalysts to psychological disturbances. Services catered to the comprehensive array of psychological, cognitive, and physical problems of the elderly have to be provided. The only reliable morbidity data is on dementia and its estimated prevalence is 33.9 per 1000 in the rural and 33.6 per 1000 in the urban population, above 60 years. Of late, 10/66 dementia research groups have contributed to our understanding of the prevalence, caregiver burden, and service delivery of dementia subjects. The Departments of Geriatric Mental Health at Lucknow, NIMHANS at Bangalore, BYL Nair Hospital at Mumbai, and a private psychiatric center at Varanasi have to be congratulated in their efforts on geriatric care.[32–35]

The geriatric specialty wing of IPS should organize a focused workshop, to help psychiatrists in the identification, assessment, and care of the elderly.

### 9. Addiction psychiatry

The World Health Organization (WHO) estimates suggest that there are 60 to 70 million alcoholics in India, of which 50% are “hazardous drinkers” and require treatment. The age of initiation to alcohol has come down from 19 years in 1986 to 13.5 years in 2006. Studies have revealed that the revenue generated from the industry (216 billion) is less than the revenue lost due to alcohol-related health problems (244 billion). In a report for WHO, a multicenter collaborative study — ‘Injury and Alcohol’ — at NIMHANS Bangalore, found that the proportion of injuries 'linked' to alcohol use was 58.9% of all injuries. Alcohol-related injuries include road accidents (46%0, violence (24%), falls, (24%) and others (6%).[36,37]

Kerala leads in per capita consumption of liquor at a whopping 8.3 liters [pushing Punjab to second place (7.9 liters)] as against four liters in the rest of the country. Kerala has been emerging as one of the largest consumers of alcohol in the world. The sale of Indian Made Foreign Liquor (IMFL) in Kerala has jumped to Rs 3,669.49 crore during 2007 – 2008, from a mere Rs 81.42 crore in 1987 – 1988. The state had received revenue of Rs 2,914.28 crore from the KSBC (Kerala State Beverages Corporation) alone as its share, besides crores of rupees from bar licenses. Kerala, the most literate of Indian states, often quoted as ‘Gods own country’ ranks high in suicide rates, accidents, and alcoholism. One really wonders whether ‘God's own country’ will be swallowed by the devil's advocate — alcoholism.[36,38,39]

Addiction to heroin (1 million), abuse of cannabis / cannabis products, psychotropics, and other ‘over-the-counter’ (OTC) drugs delineate unbridled regulation at sale outlets, with inefficient legal measures.

The addiction specialty section, I hope, will pay more attention in training psychiatrists on substance use disorders, educate the society on the evils of drug abuse, and advice the policy planners.

### 10. Undergraduate psychiatry

It is a paradox that the undergraduate (graduate) medical curriculum has lesser provision (hours of training) for psychiatry than the nursing curriculum. Despite repeated efforts to provide more thrust on Psychiatry in graduate medical education, the status quo remains, so as to contradict the WHO definition of health. With the current training, medical graduates trained in “body medicine” will be miserable in identifying common mental disorders. The medical council of India (MCI) has to intervene urgently to include psychiatric training (doctor – patient relationship, identification of common psychiatric problems, co-morbidity of psychiatric disorders in physical illness, somatic symptoms and their relevance, and basic nonpharmacological and pharmacological principles). If not, the efforts of empowering primary care providers will be a disaster. Undergraduate psychiatric training will also minimize irrational psychotropic use and ‘doctor-shopping’ of hapless subjects, with unexplained physical symptoms.

The IPS task force, on advocacy, should take immediate steps to ‘psychoeducate’ the agencies concerned.

### 11. Disaster management

India has been traditionally vulnerable to natural disasters on account of its unique geo-climatic conditions. About 60% of the landmass is prone to earthquakes of various intensities; over 40 million hectares is prone to floods; about 8% of the total area is prone to cyclones, and 68% of the area is susceptible to drought. United Nations General Assembly, in 1989, declared the decade 1990 – 2000 as the International Decade for Natural Disaster Reduction. Major natural disasters include the super cyclone in Orissa, the earthquake in Lathur and Gujarat, and the tsunami in Tamil Nadu.[40]

In recent times, many man-made disasters, as a part of terrorism, have driven the Indian society to a state of panic. The bomb blasts in Mumbai, Hyderabad, Jaipur, Delhi, Bangalore, and Guwahati have inflicted considerable psychological trauma.

In 2003 the Home Ministry has launched an India Disaster Resource Network (IDRN) and released a Status Report on Disaster Management in India in 2004. There was a suggestion for introducing emergency health management in the MBBS curriculum and in-service training of Hospital Managers and professionals. However, the involvement of psychiatric professionals either in crisis management or in managing psychological sequelae is not highlighted.

IPS has already got a taskforce on disaster management. The society has to be involved in disaster management programs of the country.

## NETWORKING WITH OTHER ORGANIZATIONS

In this century of the world becoming smaller and smaller with a pledge of “brain circulation in psychiatry” (instead of brain drain and brain gain) it is important to have networking with like-minded organizations, national and international, for better psychiatric care. This will also facilitate faculty exchanges and research collaborations.[41]

IPS is a member of the WPA (World Psychiatric Association) and the South Asian Association for Regional Cooperation (SAARC) psychiatric federation. The SAARC psychiatric federation comes under the Asian Federation of Psychiatric Association (AFPA). IPS has links with the Indo-American Psychiatric Association, Indo-Australian Psychiatric Association, British Indian Psychiatric association, and Indo-Canadian Psychiatric Association. The South Asian Forum International (SAFI), is another organization involved in training and service delivery, representing 18 countries.

The collaboration and the goodwill of these bodies should be encouraged for the benefit of Indian Psychiatry. The Royal College of Psychiatrists can assist in teaching and training, in collaboration with IPS.

I hope the networking will open new avenues of teaching and research in India.

“*You are where you are today because you stand on somebody's shoulders. And wherever you are heading, you cannot get there by yourself. If you stand on the shoulders of others, you have a reciprocal responsibility to live your life so that others may stand on your shoulders. It's the quid pro quo of life. We exist temporarily through what we take, but we live forever through what we give*”

**- Vernon Jordan**

## CONCLUSION

I have tried to outline certain areas that need special attention by the psychiatric fraternity. I shall try to initiate the process with your cooperation, but it is up to you all to carry forward the mission in the years to follow. Let us hope that IPS will take the leadership in mental health matters of India.

“When looking at the future, the “what” is far more predictable than the “when.” And the “how” will always feel different than predicted.”

**- Thomas Frey**
